# Venous Drainage Patterns in Carotid Cavernous Fistulas

**DOI:** 10.1155/2014/760267

**Published:** 2014-01-30

**Authors:** Ayse Aralasmak, Kamil Karaali, Can Cevikol, Utku Senol, Timur Sindel, Huseyin Toprak, Huseyin Ozdemir, Alpay Alkan

**Affiliations:** ^1^Department of Radiology, Bezmialem Vakif University, 34093 İstanbul, Turkey; ^2^Department of Radiology, Akdeniz University, Antalya, Turkey

## Abstract

*Purpose*. The carotid-cavernous fistula (CCF) is an abnormal arteriovenous communication and its drainage pathways may affect the clinic presentation and change treatment approach. We evaluated drainage patterns of CCFs by digital subtraction angiography (DSA) and categorized drainage pathways according to their types and etiology. *Materials and Methods*. Venous drainage patterns of 13 CCFs from 10 subjects were studied and categorized as anterior, posterior, superior, inferior, and contralateral on DSA. Drainage patterns were correlated to types and etiology of CCFs. Diagnosis of CCFs was first made by noninvasive imaging techniques. *Results*. On DSA, traumatic CCFs were usually high flow, direct type while spontaneous CCFs were usually slow flow, indirect type. Bilaterality and mixed types were observed among the indirect spontaneous CCFs. In all CCFs, anterior and inferior drainages were the most common. Contrary to the literature, posterior and superior drainages were noted only in high flow and long standing direct fistulas. Contralateral drainage was not observed in all, supporting plausible compartmentalization of cavernous sinuses. *Conclusion. *Types, etiology, and duration of the CCFs may affect their drainage patterns. DSA is valuable for categorization of CCFs and verification of drainage patterns. Drainage pathways may affect the clinic presentation and also change treatment approach.

## 1. Introduction

The CCF is an abnormal arteriovenous connection between the carotid artery and the venous cavernous sinus. CCFs are classified as direct or indirect, traumatic or spontaneous, and high or slow flow based on arterial supplies, etiology, and shunt flow rate, respectively [1]. The most commonly adopted classification is described by Barrow based on arterial supply. Direct CCF (Type A) usually occurs in young men secondary to trauma. Indirect CCFs (dural CCF) usually occur in postmenopausal, hypertensive women arising from dural branches of either internal carotid artery (ICA) (Type B) or external carotid artery (ECA) (Type C) or both (mixed or Type D) [1]. CCFs drain toward anterior via ophthalmic veins, inferior via pterygoid plexus and inferior petrosal sinus (IPS), contralateral via intercavernous connections, posterior via deep venous system, superior petrosal sinus (SPS), and cerebellar veins, and superior via superficial middle cerebral vein (SMCV). Mostly patients present with orbital symptoms secondary to anterior drainage but clinical presentation may change according to venous drainage pattern. Less commonly, headache, altered mental status, and other neurological deficits such as ischemia or infarction secondary to venous hypertension or steal phenomena may occur [2–7]. Urgent treatment is usually needed for direct and high flow fistulas in which endovascular embolization is mostly applied. Indirect, slow flow CCFs usually close spontaneously without treatment [1, 2, 5, 7]. In this study, we evaluated their venous drainage patterns by DSA and categorized their drainage patterns according to their types and etiology.

## 2. Materials and Methods

DSA views of 13 CCFs from 10 subjects were taken into consideration for categorization of venous drainage pathways. All cases were collected from single medical centre, in 10 years period. Ages of subjects were ranging from 34 to 72 and of them 5 were men and 5 were women. Two neuroradiologist (Ayse Aralasmak and Timur Sindel) evaluated the DSA views with a consensus reached in all. Diagnosis of CCFs was first made by noninvasive imaging techniques such as MRI, MR angiography (MRA), CT, CT angiograpy (CTA), or Doppler ultrasonography (USG).

## 3. Results

**Figure 1:**
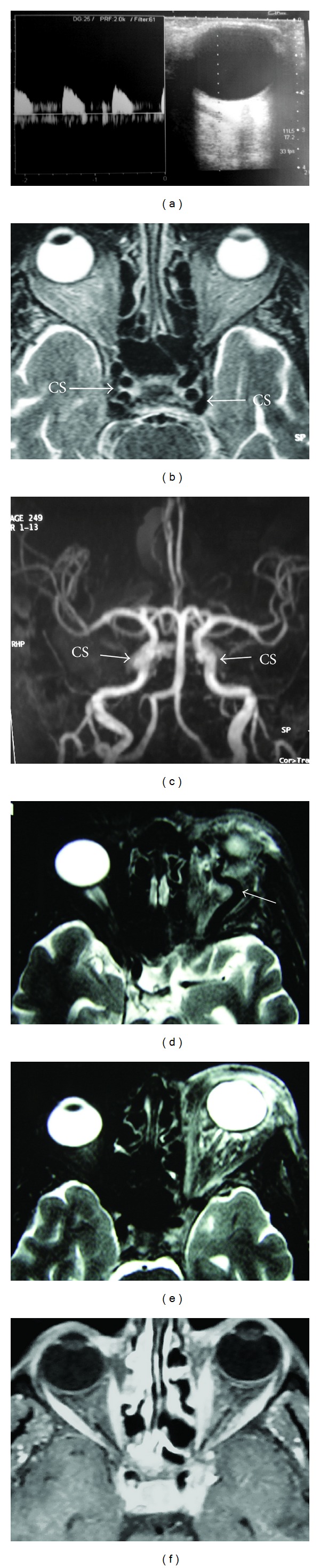
Imaging findings of CCF in different patients. (a) Arterialized flow in SOV on Doppler USG. ((b) and (c)) Arterialization of cavernous sinuses (CS) with flow void appearance on T2 weighted MRI (b) and flow related signal on maximal intensity projection MRA (c) in the same patient. ((d) and (e)) Left orbital congestion and exophthalmus with dilated SOV (arrow on d) and no evidence of flow void appearance of the left CS on T2 weighted MRI (e) but asymmetric intense enhancement of left CS (not shown here) in the same patient. (f) Asymmetric intense enhancement of left CS with no evident exophthalmus of left orbit nor arterialization of left CS on T2 weighted MRI or MRA but only dilated SOV (not shown here).

**Figure 2:**
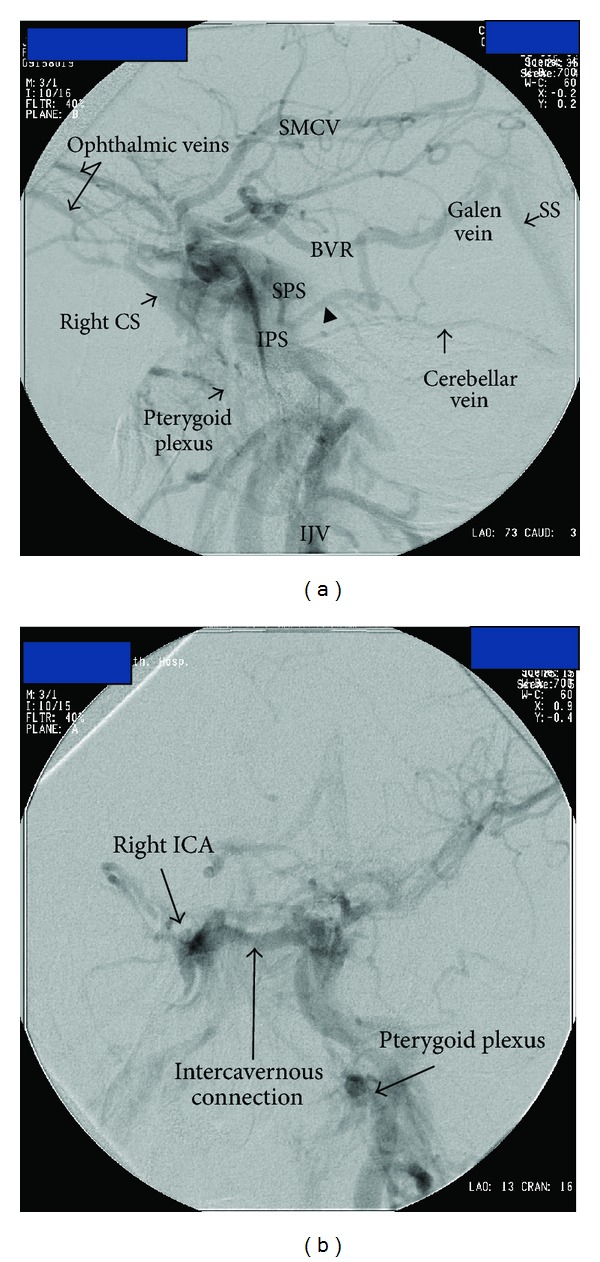
Lateral (a) and anteroposterior (b) DSA views of a posttraumatic 46-year-old male subject shows a left sided high flow direct Type A CCF with major drainage toward posterior via BVR, SPS, and cerebellar vein; superior via SMCV; inferior via IPS and minor drainage toward anterior via ophthalmic veins and contralateral via intercavernous connections. Arrowhead on (a) indicates the lateral mesencephalic vein draining from SPS into BVR. There is filling of the right CS and right pterygoid plexus via intercavernous connections. BVR: Basal vein of Rosenthal, CS: cavernous sinus, IJV: internal jugular vein, IPS: inferior petrosal sinus, SMCV: superficial middle cerebral vein, SPS: superior petrosal sinus.

**Figure 3:**
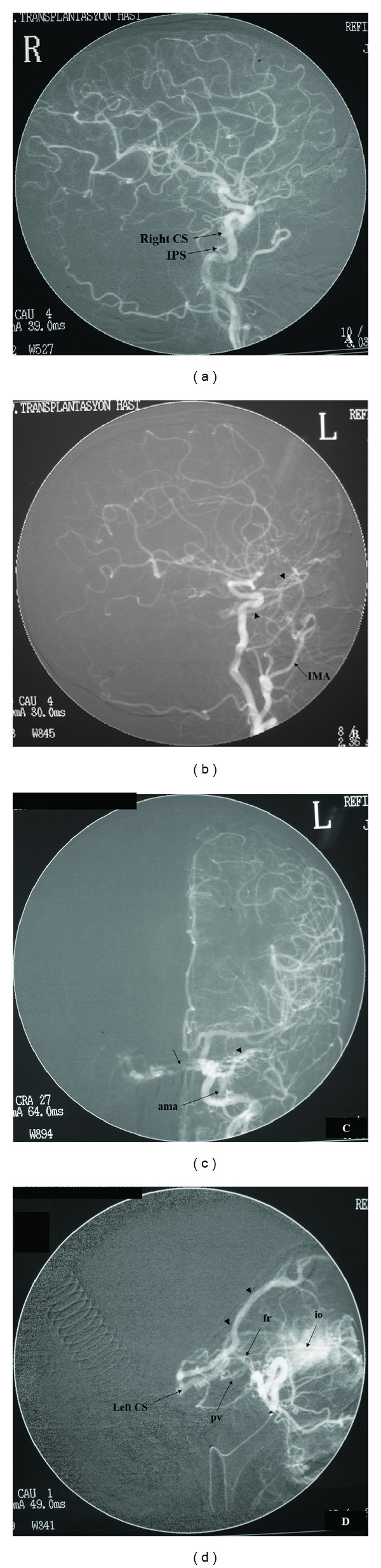
Bilateral CCFs are shown in a 50-year-old female subject with no history of trauma or aneurysm. In (a), a right sided slow flow indirect Type B CCF is filling from dural branches of right ICA with only drainage via IPS. No filling noted during selective right ECA catheterization (not shown here). In ((b), (c), and (d)), a left sided slow flow indirect Type C CCF is filling mainly from foramen rotundum (fr) and pterygovaginal (pv) branches of internal maxillary artery (IMA) and partly from the accessory meningeal artery (ama). Selective IMA catheterization (d) demonstrates the fistula better. Major drainage is toward anterior via the superior ophthalmic vein (arrowheads in (b), (c), and (d)) and minor drainage toward contralateral via intercavernous connections (small arrow in (c)). CS: cavernous sinus, IPS: inferior petrosal sinus, io: infraorbital branch of IMA.

**Figure 4:**
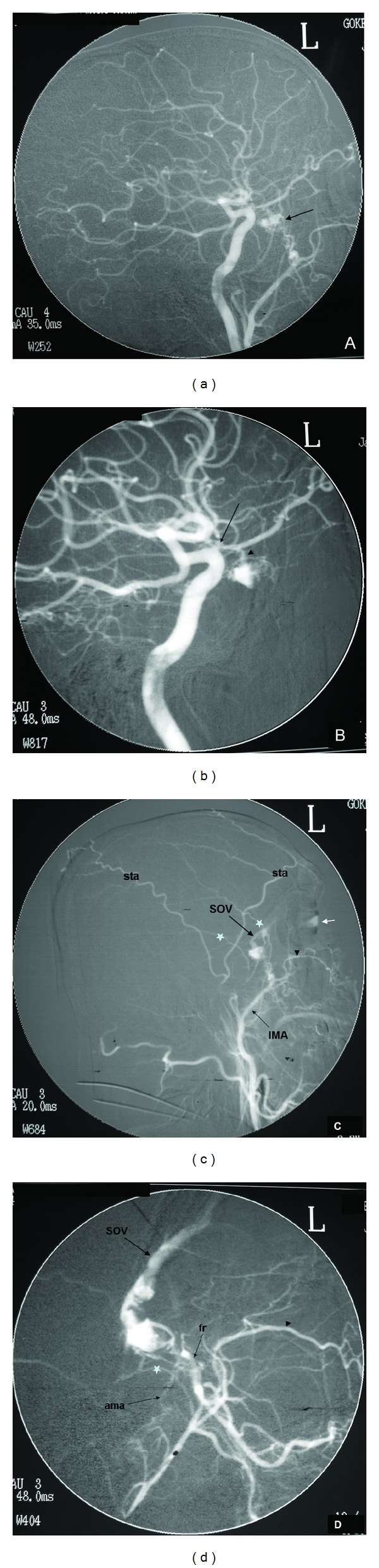
DSA views ((a), (b), (c), and (d)) of a 34-year-old male patient show a left sided, slow flow, indirect, mixed, Type D CCF. No underlying reason found for the fistula. The CCF (arrow in (a)) is fed by branches of both ICA and ECA with drainage via superior ophthalmic vein (SOV) (c) and (d)). Selective ICA catheterization (b) shows the filling of anterior aspect of the left cavernous sinus from the ophthalmic artery (arrowhead). The stenosis at the proximal aspect of the ophthalmic artery (arrow in (b)) suggests the presence of fistula for a long period of time. Selective ECA catheterization (c) and magnification (d) show filling of the fistula mainly from the foramen rotundum (fr) branch of internal maxillary artery (IMA) and minimally from accessory meningeal artery (ama). Corneal blushing is demonstrated (small white arrow in (c)). sta in (c): superficial temporal artery; stars (*) in (c) and (d): middle meningeal artery branches; black arrowheads in (c) and (d): infraorbital branch of IMA.

Findings suggestive of CCFs on noninvasive diagnostic techniques were orbital congestion, exophthalmus, dilation of SOV, asymmetric enhancement of the cavernous sinus and surrounding venous system, and arterialization of the surrounding venous structures appearing as flow void on MRI or flow related signal on MRA or early filling on CTA or reverse and pulsatile flow within the SOV on Doppler USG (Figure 1). Among 13 CCFs, direct or indirect, high or slow flow, and posttraumatic or spontaneous ones were present. Etiology, types, and drainage pathways of CCFs were shown in Table 1. Examples of CCF types with various drainage patterns were given at Figures 2, 3, and 4. CCFs were categorized according to their drainage patterns and types (Table 2). Traumatic CCFs were usually high flow, direct type. Spontaneous CCFs were usually slow flow, indirect type. Bilaterality and mixed types were observed in indirect spontaneous CCFs. In both direct and indirect CCFs, anterior and inferior drainages were the most common. Posterior and superior drainages were noted only in high flow and long standing direct fistulas. In spontaneous indirect CCFs, drainage was toward inferior, anterior, and contralateral in decreasing order. Contralateral drainage is not observed in all CCFs. In one of our cases with indirect-Type B CCF, main drainage was contralateral toward the opposite superior ophthalmic vein and minor drainage was inferior via IPS on the same side.

## 4. Discussion

CCFs could be traumatic or iatrogenic or spontaneous in etiology. Traumatic CCFs are usually high flow and direct type fistulas with sudden onset of symptoms. They predominantly occur in young men because of higher incidence of trauma in this population. Underlying mechanisms are direct injury from the skull base fracture or injury from torsion or stretching of the carotid siphon upon impact and impingement of the vessel on bony prominences. Direct CCFs following surgical procedures such as endoscopic nasal surgery and vascular neurosurgery or spontaneously from aneurysm rupture have also been reported. Spontaneous CCFs occur secondary to hypertension, atherosclerosis, neurofibromatosis, and collagen vascular disorders. They are usually slow flow indirect type fistulas with insidious onset, commonly seen in elderly, postmenopausal, and hypertensive women with another peak of incidence during pregnancy [1, 2, 7–9]. Direct CCFs occur three times as often as the indirect types [2]. Trauma is the main cause of direct CCFs [2]. Bilateral CCF cases comprise % 12–15 of all and are usually indirect in variety [2].

In our small study group, subjects with direct high flow CCFs were all men with a previous history of trauma and subjects with indirect CCFs were mostly elderly women usually with no known disease except one with a history of hypertension. Bilaterality was noted among the indirect CCFs [1, 2]. Our findings so far are in accordance with the literature. In our study group, drainage pathways were toward anterior via ophthalmic veins, toward inferior via pterygoid plexus and IPS, toward contralateral via intercavernous connections, toward posterior via deep venous system (Basal vein of Rosenthal (BVR)), SPS, and cerebellar veins, and toward superior via SMCV. In both direct and indirect CCFs, anterior and inferior drainages were the most common routes. However, posterior and superior drainages were noted only in long standing high flow direct CCFs. In spontaneous indirect CCFs, inferior, anterior, and contralateral drainages were observed in decreasing frequency. Contralateral drainage was not observed in all CCFs suggesting the possibility of compartmentalization within the cavernous sinuses.

Cavernous sinuses are composed of small venous compartments. It is accepted that these compartments are joined by several anastomoses across the midline. We thought that all compartments were not connected to each other (compartmentalization) since in cases of unilateral CCF, contralateral drainage was not observed in all.

In CCF, venous drainage patterns and shunt flow rate influence the clinical presentation. Anterior drainage induces orbital congestive symptoms. Posterior and inferior drainage may induce cranial nerve palsies, probably due to neural compression by an expanding cavernous sinus or IPS, or venous congestive changes in the posterior fossa and brain stem [2, 5–7, 10]. High flow shunt may cause ischemia or arterial infarct due to steal phenomena [3, 5–7]. Spontaneous CCFs may present with atypical clinical findings, such as ocular signs contralateral to the fistula side or cranial nerve palsy only [5]. Posterior and superior drainages may have significant results such as seizure, congestion or venous infarctions in the cerebrum, cerebellum, deep brain structures and brain stem [4, 6, 7]. BVR, a part of deep venous and posterior drainage pathway, may drain into the Galenic vein, cavernous sinus, and sphenoparietal sinus (SPS) via the peduncular and lateral mesencephalic veins and into transverse sinus or straight sinus via the tentorial veins [11]. Drainage via BVR in our study was only noted in high flow direct CCFs. Lateral mesencephalic vein, a draining vein of brain stem, was noted in the direct CCF (Figure 2), connecting the BVR to SPS [12]. Superior drainage via SMCV may result in cortical venous infarct. The SMCV is a cortical vein connecting to the sphenoparietal or cavernous sinus (60%), or to the pterygoid plexus (14%) in normal population [13]. It may also drain into transverse sinus through the vein of Labbe or superior sagittal sinus through the vein of Trolard [14]. The SMCV was noted in our two subjects with long standing high flow direct CCFs draining into the superior sagittal sinus via Trolard vein (Figure 2).

There is somewhat controversy when comparing our results to the literature. We did not find any superior or posterior drainage in our cases with indirect CCFs, but Suh et al. observed superior drainage in 21%, posterior drainage in 15%, and both posterior and superior drainage in 5% of total 58 cases (50 females, 8 males) with indirect CCFs [6]. In the larger series of indirect CCFs, Kirsch et al. reported superior and deep venous drainage in 34%, anterior drainage via the SOV in 88%, inferior drainage via the IPS in 42%, and contralateral drainage via intercavernous connections in 23% of total 141 cases (121 females, 20 males) [7]. Absence of posterior and superior drainage within our cases of indirect fistula may be the result of the limited patient population or related to time onset of fistulization since posterior and superior drainages were noted only in high flow direct CCFs existing for a long time.

In diagnosis and classification of CCFs, DSA is still a gold standard although findings from noninvasive imaging techniques are helpful for diagnosing. These are uni- or bilateral exopthalmus, orbital congestion, dilation of SOV, arterialization of the surrounding venous structures appearing as flow void on MRI or flow related signal on MRA or early filling on CTA or reverse flow or arterialized flow within the SOV on Doppler ultrasonography, and asymmetric enhancement of cavernous sinuses and surrounding venous system [15, 16] (Figure 1). Flow void appearance on T2 weighted images depends on the flow rate within the cavernous sinus and veins. Evaluation of the drainage patterns of CCFs may help planning the endovascular treatment [7]. In direct CCFs, either transarterial or transvenous embolization of the fistula site is usually performed. Indirect dural CCFs have a relatively high incidence of spontaneous resolution (10–73%); therefore, invasive diagnostic measures and treatment might not be necessary but only manual ICA compression [1, 2, 5, 7]. Among endovascular treatment of direct CCFs and symptomatic indirect CCFs, transarterial embolization is a more common approach. Transvenous embolization is employed in the presence of multiple arterial feeders and inability to occlude indirect CCFs by the arterial route. The inferior petrosal sinus is the most common route in transvenous approach. If the sinus is impassable, alternative routes are the pterygoid venous plexus, superior petrosal sinus, facial vein, or ophthalmic veins [17].

## 5. Conclusion

Although noninvasive radiologic techniques can aid diagnosis of the CCFs, DSA is still a gold standard for diagnosis and valuable for categorization of CCFs and verification of their drainage patterns. Etiology, type, and duration of the CCFs may affect their drainage patterns. Drainage pathways may affect the clinic presentation and change treatment approach. In our small study group of CCF, main drainage in all was toward anterior and inferior. Contrary to the literature, posterior and superior drainages were noted only in long standing high flow direct CCFs. Contralateral drainage was not observed in all, supporting the possibility of compartmentalization within the cavernous sinuses.

## Figures and Tables

**Table 1:** Etiology, types, and drainage patterns of subjects with CCF are shown.

CCF	Subject	Gender, age	Type of CCF	Etiology	Major drainage	Minor drainage
1	1 left CCF	M, 46	Direct-high flow-Type A	Traumatic	Inferior, posterior, superior	Anterior, contralateral
2	2 right CCF	F, 65	Indirect-slow flow-Type B	Spontaneous	Anterior, contralateral	Inferior
3	2 left CCF	F, 65	Indirect-slow flow-Type B	Spontaneous	Anterior, contralateral	Inferior
4	3 left CCF	M, 40	Direct-high flow-Type A	Traumatic	Anterior	Inferior
5	4 right CCF	M, 49	Direct-high flow-Type A	Traumatic	Anterior	Posterior, inferior, superior
6	5 left CCF	M, 16	Direct-high flow-Type A	Traumatic	Anterior	Contralateral, inferior, posterior
7	6 right CCF	F, 50	Indirect-slow flow-Type B	Spontaneous	Inferior	—
8	6 left CCF	F, 50	Indirect-slow flow-Type C	Spontaneous	Anterior	Contralateral
9	7 left CCF	M, 34	Indirect-slow flow-mixed-Type D	Unknown or spontaneous	Anterior	—
10	8 left CCF	F, 62	Indirect-slow flow-mixed-Type D	Spontaneous, hypertension	Inferior	—
11	9 left CCF	F, 72	Indirect-slow flow-Type B	Spontaneous	Anterior	Contralateral, inferior
12	10 right CCF	F, 61	Indirect-slow flow-Type B	Spontaneous	Contralateral	Inferior
13	10 left CCF	F, 61	Indirect-slow flow-Type B	Spontaneous	Anterior	Inferior

**Table 2:** Types of CCFs are categorized according to frequency of their drainage pathways.

Type of CCF	Drainage pathway				
	Anterior	Inferior	Contralateral	Posterior	Superior
Type A	4	4	2	3	2
Type B	4	6	4	0	0
Type C	1	0	1	0	0
Type D	1	1	0	0	0
